# Redo cases for mitral valve replacement on fibrillating heart – is the outcome better?

**DOI:** 10.1186/1749-8090-8-S1-O281

**Published:** 2013-09-11

**Authors:** D Petkov, V Papantchev, B Baev, G Nachev

**Affiliations:** 1 Department of Cardiac Surgery, ‘St. Ekaterina’ University Hospital, Sofia, Bulgaria

## Background

Nowadays needs for redo cardiac surgery is more common. Number of patients requiring reoperation for significant mitral regurgitation is increasing. The aim of present work is to review our experience with redo cases for mitral valve replacement on fibrillating heart.

## Methods

Between 2011 and 2013 a total number of 55 patients, 29 female and 26 male, with average age 62 years (from 42 to 75), underwent redo mitral surgery in our institution. Those were divided in two groups. Group 1 – patients operated on fibrillation heart without aortic cross-clamping (15 patients, average age 61 years, 4 female and 11 males) and Group 2 – patients operated with aortic cross-clamping and cardioplegic arrest (40 patients, average age 62 years, 25 female and 15 male). In all patients mitral valve replacement was performed. In 32 patients at least one more major cardiac procedure was also performed during the same surgery.

## Results

In hospital mortality in group 1 was 2 patients (13%) while in group 2 it was 8 patients (20%). The CPB time was shorter in group 1 (average 95 min) compared to group 2 (108 min). Complications rate were as follows: heart failure – 3 patients (20%) in group 1 versus 15 patients (37.5%) in group 2; acute renal failure – 1 patient (7%) in group 1 versus 11 patients (27.5%) in group 2; sepsis – 2 patients (13%) in group 1 versus 3 patients (7.5%) in group 2; CNS complications – 1 patients (7%) in group 1 versus 1 patients (2.5%) in group 2.

## Conclusions

Our experience shows that redo cases for mitral valve replacement on fibrillating heart have better outcome. The mortality and morbidity are reduces. We believe that the technique is easier and straightforward.

